# Supplementary material for: Incubation Time Influences Organic Anion Transporter 1 Kinetics and Renal Clearance Predictions
Source: J Xenobiot. 2023 May 10;13(2):205–17. doi: 10.3390/jox13020016 (PMC10204490; doi:10.3390/jox13020016)
Supplement: Supplementary file 1 [file jox-13-00016-s001.zip › jox-2338681-supplementary.pdf]

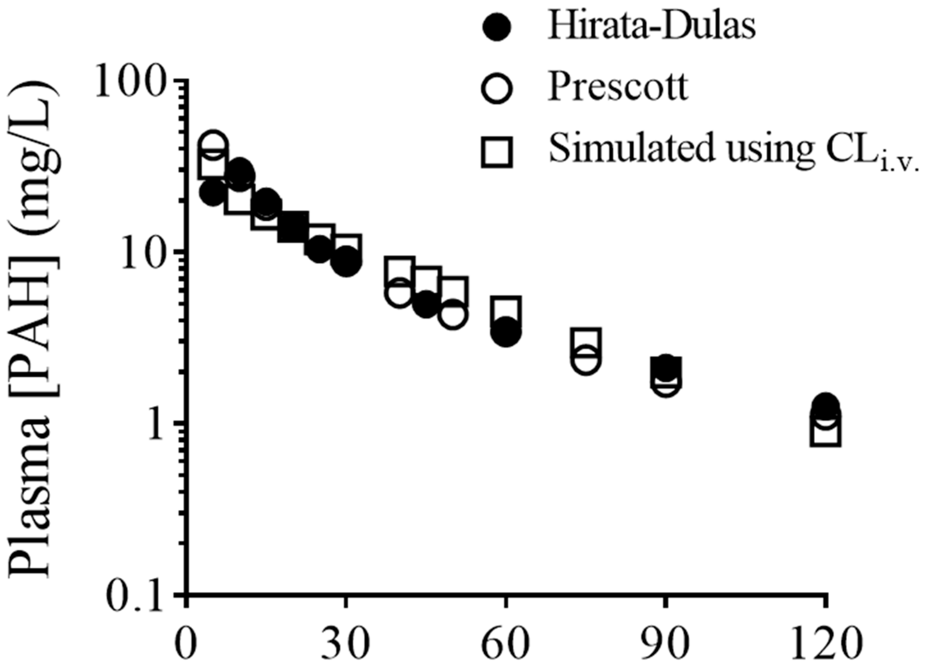

Figure S1. Pharmacokinetic predictions for PAH using its clinically observed plasma intravenous clearance value. To test the accuracy of the drug-dependent input parameters toward further building a MechKiM-PBPK model for OAT1, the first simulation used the clinically-observed plasma clearance of PAH following a 10 mg · kg^{-1} intravenous dose ($CL_{i.v.}$), which is reported as 40.6 L · h^{-1} [13]. The observed plasma concentration-time profiles for PAH are from [12,13]. Both studies administered a 10 mg/kg intravenous dose of PAH to healthy volunteers. The 'drug-dependent' parameters used for PAH were as follows. The molecular weight, LogP and *p*Ka were 194.2 g · mol^{-1}, -2.2 and 3.83 [28], respectively. We used the 'default' blood-to-plasma partition ratio (B/P) of one, and the measured fraction unbound in plasma (f_{up}) of 0.83 [28]. Using the Full PBPK model, Method 2 [29], and a K_p scalar of one, the predicted steady-state volume of distribution (V_{ss}) was 0.29 L · kg^{-1}, which approximates the observed V_{ss} of 0.23 L · kg^{-1} [14]. When using the initial drug-dependent parameters and the observed plasma $CL_{i.v.}$, the simulated and observed plasma concentration-time profiles were in good agreement with each other indicating that the drug-dependent input parameters were appropriate for further model development using MechKiM. .

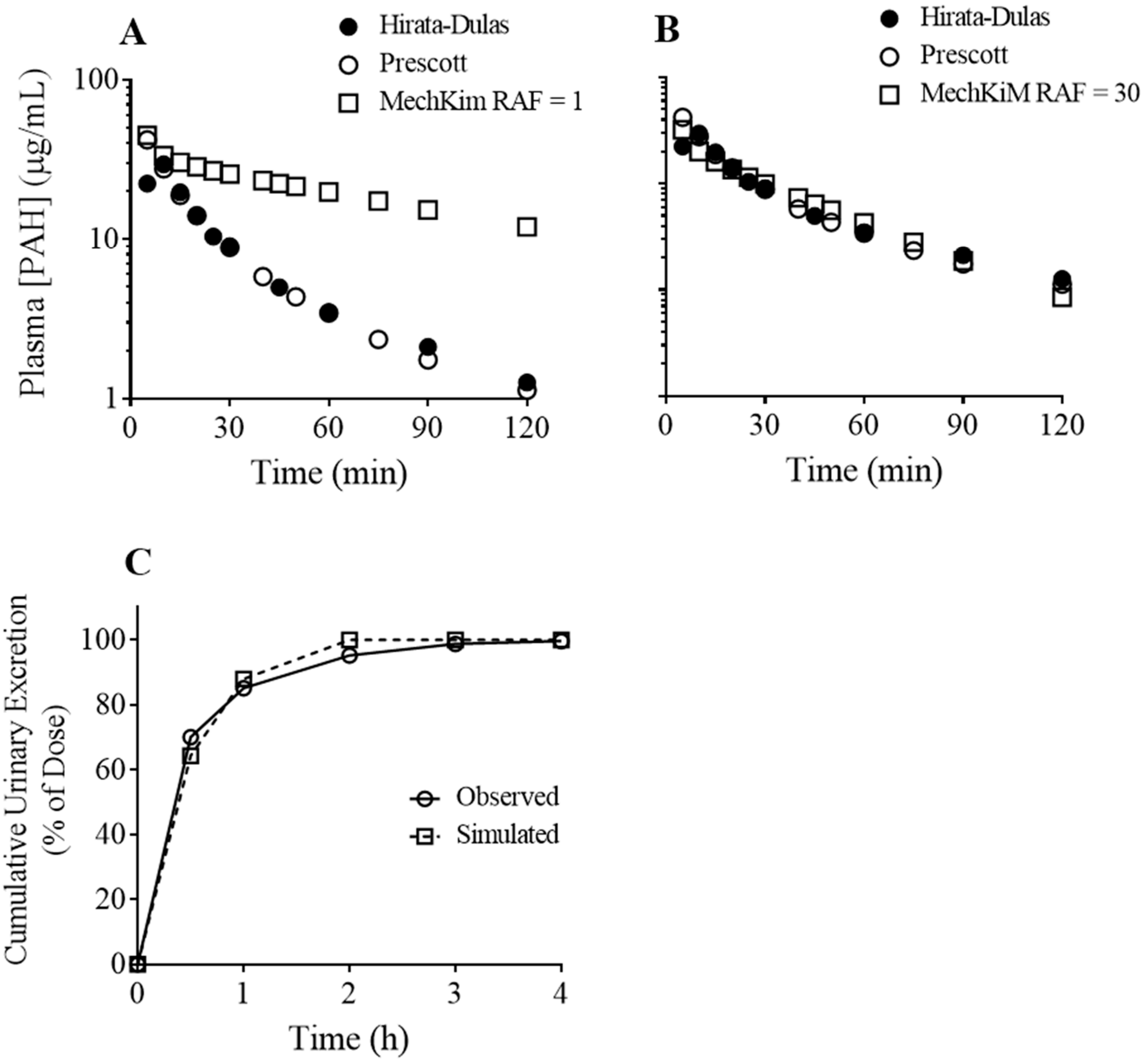

Figure S2. Development of a PBPK-MechKiM model of OAT1 involvement in renal PAH clearance. .

To develop the PBPK model for PAH using MechKiM, we made the following assumptions, with justifications to follow: (i) PAH is excreted exclusively by the kidney as the parent compound, by glomerular filtration and tubular secretion, with no other elimination pathways; (ii) OAT1 represents the only uptake step for PAH into renal tubule cells; (iii) there is transporter-mediated efflux of PAH into the renal tubule lumen; (iv) there is no appreciable passive diffusion of PAH across the basolateral or apical membranes of tubule cells, and no appreciable reabsorption occurs.

First, the majority of PAH is eliminated in the urine as the parent compound, and only a small percentage of the dose (~10%) is excreted into the urine as acetyl-PAH [12,13]. Also, acetyl-PAH is actively secreted by renal tubules, and has been suggested to compete with PAH for the secretory process – likely due to shared transport mechanisms (i.e., OAT1) [30]. That is, elevated plasma concentrations of PAH reduce the renal clearance of acetyl-PAH [30]. Second, although OAT1 and OAT3 represent the major pathways for organic anion uptake across the basolateral membrane of renal tubule cells [1], PAH is a preferred substrate of OAT1 over OAT3 [31]. Third, MRP2 and MRP4 are organic anion efflux transporters expressed in the apical membranes of renal tubule cells, which likely contribute to PAH efflux into the tubule lumen [32]. Finally, PAH is hydrophilic, and therefore, its passive diffusion across biological membranes should be negligible, as we observe in *In vitro* studies. Additionally, tubular reabsorption appears to have a limited role in the renal clearance of drugs that undergo active renal tubular secretion [13].

In the next simulations we used the MechKiM model and added OAT1 at the basolateral membrane and an efflux pathway at the apical membrane (i.e., MRP). Given the low passive permeability of PAH, we assumed the intrinsic clearance across the basolateral and apical membranes due to passive diffusion to be zero. For the OAT1-mediated intrinsic uptake clearance ($CL_{int,uptake}$), the value obtained at the 15 sec time point (5.5 µl · min^{-1} · million cells^{-1}) was used. In addition to an *in vitro* intrinsic clearance value, the MechKiM model requires input of a relative activity factor (RAF) value for *in vitro-in vivo* extrapolation (IVIVE) of transport activity. The RAF value corrects for several factors including: (i) differences in transport protein abundance *in vitro* compared to *in vivo*, (ii) *in vitro-in vivo* differences in transport energetics, and (iii) uncertainty regarding the number of tubule cells per gram kidney required for scaling *in vitro* data. Given these unknowns, we initially set the RAF value for uptake to one. Also, the intrinsic clearance ($CL_{int,efflux}$) and RAF value for the efflux pathway were set to 1 µl · min^{-1} · million cells^{-1} and one, respectively. Using these values, the simulated AUC was over-predictive of the observed AUC (**A**) [12,21], indicating an under-prediction of the simulated renal clearance of PAH. To account for this, the $CL_{int,uptake}$ was fixed to 5.5 µl · min^{-1} · million cells^{-1}, and then 'parameter estimation' was used to determine the appropriate RAF value for OAT1, in order to recover the simulated renal clearance of PAH – that value was thirty (RAF = 30). Using the $CL_{int,uptake}$ value at the 15 sec time point, and a RAF = 30, the simulated and observed plasma concentration-time profiles for PAH were in good agreement with each other (**B**). Importantly, "Sensitivity Analysis" indicated that PAH clearance, C_{max} and AUC were insensitive to 100-fold changes in $CL_{int,efflux}$ values, but very sensitive to changes $CL_{int,uptake}$ values (data not shown), indicating that the intrinsic clearance and RAF values for uptake and efflux in the final model were appropriate. Indeed, experimental evidence from several studies strongly suggest that the basolateral uptake step is the rate-determining step in renal tubular organic anion secretion [33–36]. To further support the validity of our model for its proposed use, the simulated versus observed cumulative urinary excretion-time profile for PAH when using these final MechKiM input parameters were almost exact (**C**) [14].

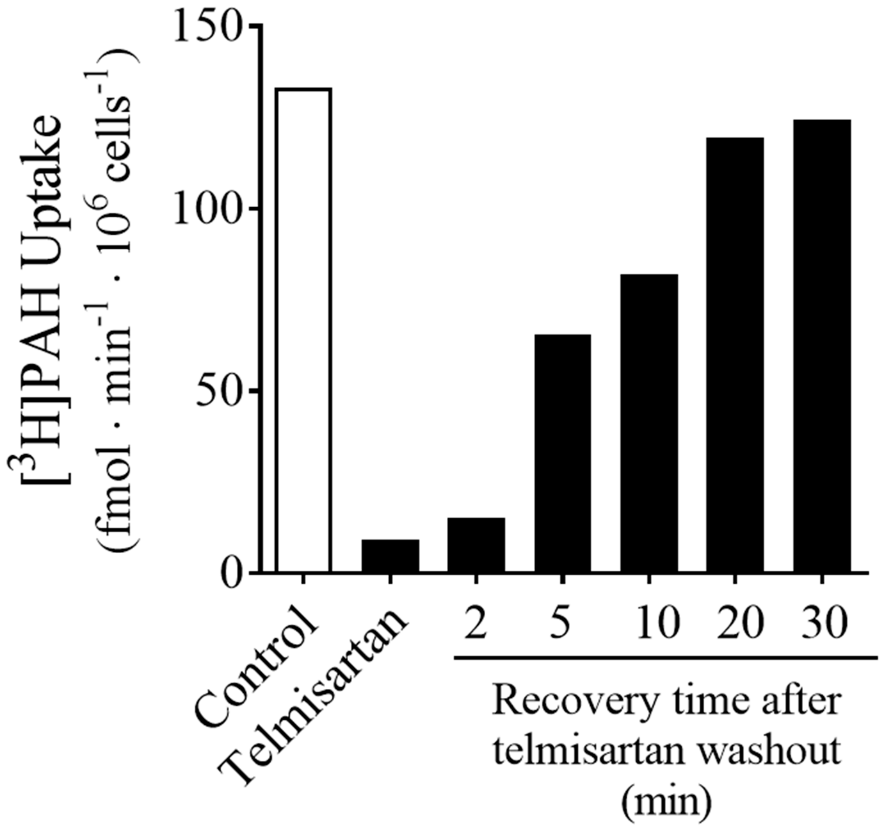

Figure S3. Time course of recovery of OAT1-mediated [^{3}H]PAH uptake by CHO-OAT1 cells after pre-treatment with telmisartan. The cells were pre-treated for 30 min with telmisartan (0.5 μM) or no telmisartan (control). Following telmisartan exposure, the cells were rinsed and incubated in Waymouth buffer containing 10% fetal bovine serum for the time points indicated (recovery time after telmisartan washout) prior to measuring the uptake of [^{3}H]PAH for 15 sec. .

Table S1. A. Effect of time on OAT1 transport kinetics.

**Incubation Time**	**J_{max}** *($pmol \cdot min^{-1} \cdot 10^6 cells^{-1}$)*	**K_m** *(μM)*	**CL_{int}** *($\mu l \cdot min^{-1} \cdot 10^6 cells^{-1}$)*
15 sec	24.9 ± 9	4.4 ± 0.6	5.5 ± 1.6
1 min	21.3 ± 7.7	4.4 ± 0.5	4.8 ± 1.6
5 min	16 ± 5.1	6.7 ± 0.7	2.4 ± 0.7
10 min	9.1 ± 1.7	7 ± 0.6	1.4 ± 0.4
30 min	6.7 ± 4.9	5.3 ± 0.4	1.2 ± 0.07
45 min	3.9 ± 2.7	6.8 ± 0.3	0.5 ± 0.3

Data are mean ± SEM of four experiments. Significant differences in kinetic parameters determined at different time points (15 sec to 45 min) were determined by One-Way ANOVA. There was a significant effect of time on all three kinetic parameters: J_{max} ($P < 0.05$), K_m ($P < 0.01$), and CL_{int} ($P < 0.01$). .

Table S1. B. Effect of time on OAT1 inhibition.

**Inhibitor**	**IC_{50}, μM** **(15 sec)**	**(10 min)**	**Fold change**
Omeprazole	9.7 ± 0.8	9.2 ± 0.8	1.1
Furosemide	19.6 ± 3	15.1 ± 2.2	1.3
Indomethacin	5.9 ± 0.5	12.7 ± 1.4**	2.2
Probenecid	9.3 ± 1.3	5.1 ± 0.6*	1.8
Telmisartan	0.4 ± 0.07	0.06 ± 0.02**	6.7

Data are mean ± SEM of four experiments. *$P < 0.05$, **$P < 0.001$, indicates significant differences in IC_{50} values between 15 sec and 10 min, unpaired Student's *t* test.

References

1. Pelis, R.M.; Wright, S.H. Renal transport of organic anions and cations. *Compr. Physiol.* **2011**, *1*, 1795–1835. https://doi.org/10.1002/cphy.c100084.

12. Hirata-Dulas, C.A.; Awni, W.M.; Matzke, G.R.; Halstenson, C.E.; Guay, D.R. Evaluation of two intravenous single-bolus methods for measuring effective renal plasma flow. *Am. J. Kidney Dis.* **1994**, *23*, 374–381. https://doi.org/10.1016/s0272-6386(12)80999-1.

13. Prescott, L.F.; Freestone, S.; McAuslane, J.A. The concentration-dependent disposition of intravenous p-aminohippurate in subjects with normal and impaired renal function. *Br. J. Clin. Pharmacol.* **1993**, *35*, 20–29.

28. Smith, H.W.; Finkelstein, N.; Aliminosa, L.; Crawford, B.; Graber, M. The renal clearances of substituted hippuric acid derivatives and other aromatic acids in dog and man 1. *J. Clin. Investig.* **1945**, *24*, 388–404. https://doi.org/10.1172/jci101618.

29. Rodgers, T.; Rowland, M. Mechanistic approaches to volume of distribution predictions: Understanding the processes. *Pharm. Res.* **2007**, *24*, 918–933. https://doi.org/10.1007/s11095-006-9210-3.

30. Dowling, T.; Frye, R.; Fraley, D.S.; Matzke, G.R. Characterization of tubular functional capacity in humans using para-aminohippurate and famotidine. *Kidney Int.* **2001**, *59*, 295–303. https://doi.org/10.1046/j.1523-1755.2001.00491.x.

31. Tahara, H.; Kusuhara, H.; Chida, M.; Fuse, E.; Sugiyama, Y. Is the monkey an appropriate animal model to examine drug-drug interactions involving renal clearance? effect of probenecid on the renal elimination of H_2 receptor antagonists. *Experiment* **2005**, *316*, 1187–1194. https://doi.org/10.1124/jpet.105.094052.

32. Smeets, P.H.; van Aubel, R.A.A.A.; Wouterse, A.C.; Heuvel, J.J.V.D.; Russel, F.G. Contribution of multidrug resistance protein 2 (MRP2/ABCC2) to the renal excretion of p-aminohippurate (PAH) and identification of mrp4 (ABCC4) as a novel pah transporter. *J. Am. Soc. Nephrol.* **2004**, *15*, 2828–2835. https://doi.org/10.1097/01.asn.0000143473.64430.ac.

33. Mathialagan, S.; Piotrowski, M.A.; Tess, D.A.; Feng, B.; Litchfield, J.; Varma, M.V. Quantitative prediction of human renal clearance and drug-drug interactions of organic anion transporter substrates using in vitro transport data: A relative activity factor approach. *Drug Metab. Dispos.* **2017**, *45*, 409–417. https://doi.org/10.1124/dmd.116.074294.

34. Chatsudthipong, V.; Dantzler, W.H. PAH-alpha-KG countertransport stimulates PAH uptake and net secretion in isolated snake renal tubules. *Am. J. Physiol.* **1991**, *261 Pt 2*, F858–F867. https://doi.org/10.1152/ajprenal.1991.261.5.f858.

35. Chatsudthipong, V.; Dantzler, W.H. PAH/alpha-KG countertransport stimulates PAH uptake and net secretion in isolated rabbit renal tubules. *Am. J. Physiol.* **1992**, *263 Pt 2*, F384–F391. https://doi.org/10.1152/ajprenal.1992.263.3.f384.

36. Watanabe, T.; Maeda, K.; Kondo, T.; Nakayama, H.; Horita, S.; Kusuhara, H.; Sugiyama, Y. Prediction of the hepatic and renal clearance of transporter substrates in rats using in vitro uptake experiments. *Drug Metab. Dispos.* **2009**, *37*, 1471–1479. https://doi.org/10.1124/dmd.108.026062.
